# Eicosanoid Diversity of Stony Corals

**DOI:** 10.3390/md16010010

**Published:** 2018-01-03

**Authors:** Helike Lõhelaid, Nigulas Samel

**Affiliations:** Department of Chemistry and Biotechnology, Tallinn University of Technology, Akadeemia tee 15, 12618 Tallinn, Estonia; nigulas.samel@ttu.ee

**Keywords:** allene oxide synthase-lipoxygenase, arachidonic acid, coral, cyclooxygenase, eicosanoids, lipoxygenase

## Abstract

Oxylipins are well-established lipid mediators in plants and animals. In mammals, arachidonic acid (AA)-derived eicosanoids control inflammation, fever, blood coagulation, pain perception and labor, and, accordingly, are used as drugs, while lipoxygenases (LOX), as well as cyclooxygenases (COX) serve as therapeutic targets for drug development. In soft corals, eicosanoids are synthesized on demand from AA by LOX, COX, and catalase-related allene oxide synthase-lipoxygenase (cAOS-LOX) and hydroperoxide lyase-lipoxygenase (cHPL-LOX) fusion proteins. Reef-building stony corals are used as model organisms for the stress-related genomic studies of corals. Yet, the eicosanoid synthesis capability and AA-derived lipid mediator profiles of stony corals have not been determined. In the current study, the genomic and transcriptomic data about stony coral LOXs, AOS-LOXs, and COXs were analyzed and the eicosanoid profiles and AA metabolites of three stony corals, *Acropora millepora*, *A. cervicornis*, and *Galaxea fascicularis*, were determined by reverse-phase high-performance liquid chromatography (RP-HPLC) coupled with MS-MS and a radiometric detector. Our results confirm that the active LOX and AOS-LOX pathways are present in *Acropora* sp., which correspond to the genomic/sequence data reported earlier. In addition, LOX, AOS-LOX, and COX products were detected in the closely related species *G. fascicularis*. In conclusion, the functional 8*R*-LOX and/or AOS-LOX pathways are abundant among corals, while COXs are restricted to certain soft and stony coral lineages.

## 1. Introduction

Oxygenated polyunsaturated fatty acids (PUFAs), oxylipins, are well-defined stress mediators in animals and plants. Their formation is initiated by fatty acid dioxygenases-lipoxygenases (LOX) [1] and cyclooxygenases (COX) [2]. Main substrates for LOXs in animals and plants are C20 (arachidonic acid, AA) and C18 (α-linolenic and linoleic acids) PUFAs, respectively [3]. The metabolites of eicosanoid pathways are widely used as drugs and dioxygenases as drug targets [4]. Eicosanoids act as local hormones in auto- or paracrine manner. They control inflammatory processes, pain, fever, cancer, and neural disorders [5,6,7]. Eicosanoid producing dioxygenases are up-regulated/activated in pathophysiological conditions and their inhibition by drugs provides relief [8,9,10]. Multiple drugs, e.g., nonsteroidal anti-inflammatory drugs, including selective COX-2 inhibitors, 5-LOX and 12/15-LOX inhibitors, and leukotriene receptor antagonists [4,9,11], have been developed to reduce pain, fever, and inflammation. All together constituting dioxygenases as therapeutic targets for a continuing extensive drug development.

Historically, certain corals have been the richest natural source for optically active prostaglandins (PG). The first coral eicosanoid studies resulted in the detection of large quantities of PGs and PG-esters (2–3% by dry weight) in the soft coral *Plexaura homomalla* [12]. Thereafter, a plethora of eicosanoids which vary depending on species and location were discovered [13,14]. In soft corals, AA is an abundant fatty acid (10–25%), being the primary precursor of eicosanoids [15,16]. Free AA is metabolized by COX [17,18,19] or LOX [20] into PGs or hydro (peroxy)-eicosatetraenoic acids (H(p)ETE), respectively. In addition to 11*R*-LOX [20], a catalase-related allene oxide, synthase-8*R*-lipoxygenase (cAOS-LOX) [21,22], and hydroperoxide lyase-8*R*-lipoxygenase (cHPL-LOX) [23,24] fusion proteins contribute to the production of different eicosanoids in soft corals. Furthermore, the AOS-LOX pathway is involved in mechanical and thermal stress response [25,26].

Stony corals are the foundation of coral reefs. Global climate warming and environmental stressors have emerged as major threats to the survival of coral reefs [27]. *Acropora* sp. is widely used as a model organism in the transcriptomic stress studies of stony corals [28,29,30,31,32,33,34,35,36,37]. Although the presence of multiple eicosanoid pathways, e.g. AOS-LOX and 5-LOX, in stony corals has been proposed by several comparative transcriptomic studies [38,39], the content of eicosanoids in these corals has not yet been determined. AA also contributes to the fatty acid content of stony corals, accounting for 3–11% of total PUFA content [40,41,42]. Thus, indeed, all the proposed pathways and metabolites could be present in stony corals. Although COX orthologs are present in many lineages of life, from algae [43] and crustaceans [44] to mammals [45], the analysis of coral transcriptomic data confirms that COXs are present only in octocorals and not in hexacorals [46]. To date, no study on stony coral (AOS)-LOX sequences has been reported and only one AOS, which is present in *A. palmata* and belongs to the plant CYP74 family of cytochrome P450 superfamily, has been characterized using the C18 PUFA substrate [47].

Based on the above, we predicted that the model organism *Acropora* sp. would contain the activity and metabolites of AOS-LOXs and various LOXs, e.g., 5-, 8-hydroxyeicosatetraenoic acid (HETE), and leukotrienes (LTs), but not the activity of COX or PGs. *Galaxea fascicularis* was included in the analysis to test the variance of eicosanoid profiles between stony corals. In this study, we analyzed the available sequence data on stony coral dioxygenases, determined the enzymatic activity of AA metabolizing enzymes, and identified endogenous eicosanoids isolated from stony corals *A. millepora*, *A. cervicornis*, and *G. fascicularis*.

## 2. Results

### 2.1. Stony Coral Dioxygenases

#### 2.1.1. Lipoxygenases

**Figure 1:**
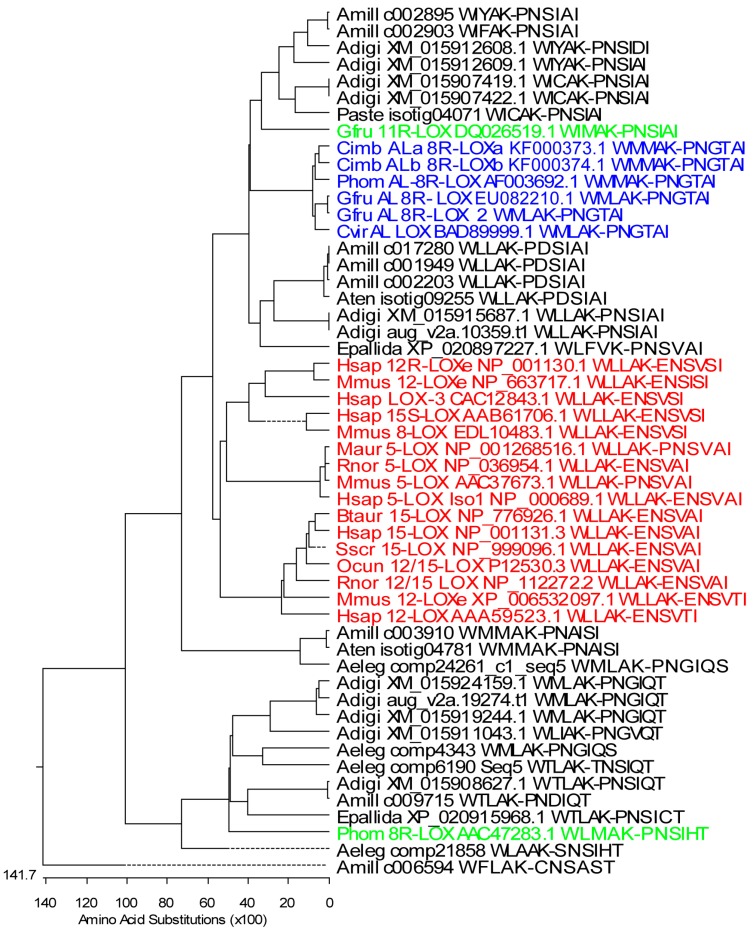
Phylogenetic tree of partial coral and mammalian LOXs. Only sequences containing WLLAK to PNSIAI (about 318–330 aa) were included in the analysis. The maximum-likelihood tree was created by Lasergen MegAlign (DNASTAR, Inc., Madison, WI, USA) Stony coral LOXs: *A. digitifera*, Adigi: XM_015907419.1, XM_015907422.1, XM_015908627.1, XM_015911043.1, XM_015912608.1, XM_015912609.1, XM_015915687.1, XM_015919244.1, XM_015924159.1, aug_v2a.10359.t1, and aug_v2a.19274.t1; *A. millepora*, Amill: c017280, c003910, c009715, c006594, full-length AOS-LOXs: c002895, c002903, c001949, and c002203; *A. tenuis*, Aten: isotig04781 and isotig09255; *P. astreoides*, Paste: isotig04071; *Anthopleura elegantissima*, Aeleg: comp4343, comp6190_c0_seq5, comp21858, comp24261_c1_seq5; *Exaiptasia pallida*, Epallida: XP_020897227.1 and XP_020915968.1. Soft coral LOXs: *G. fruticosa*, Gfru: AOS-8*R*-LOX and AOS-8R-LOX2 (EU082210.1 and personal data), 11*R*-LOX (DQ026519.1); *C. imbricata*, Cimb: AOS-8*R*-LOXa and HPL-8*R*-LOX (KF000373 and KF000374); *P. homomalla*, Phom: AOS-8*R*-LOX (AF003692.1) and 8*R*-LOX (AAC47283.1), and *Clavularia viridis*, Cvir: putative AOS-LOX (AB188528.1). Mammalian LOXs: *Bos taurus*, Btaur: 15-LOX (NP_776926.1); *Homo sapiens*, Hsap: 5-LOX (NP_000689.1), 12-LOX(AAA59523.1), 15-LOX (NP_001131.3), 15S-LOX(AAB61706.1), LOX-3 (CAC12843), 12*R*-LOXe (NP_001130.1); *Mesocricetus auratus*, Maur: 5-LOX (NP_001268516.1); *Mus musculus*, Mmus: 12*R*-LOXe (NP_663717.1), 12/15-LOX XP_006532097.1), 5-LOX (AAC37673.1), 8-LOX (EDL10483.1); *Oryctolagus cuniculus*, Ocun: 12/15-LOX (P12530.3); *Rattus norvegicus*, Rnor: 5-LOX (NP_036954.1), 12/15-LOX (NP_112272.2); *Sus scrofa*, Sscr: 15-LOX (NP_999096.1). Black—stony coral LOXs, blue—soft coral AOS-LOXs, green—soft coral LOXs, red—mammalian LOXs.

**Figure 2:**
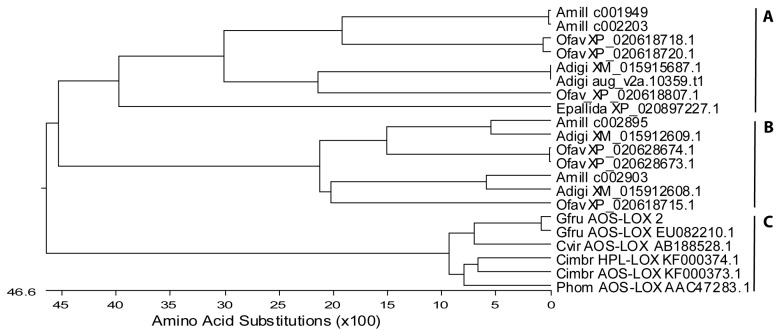
Phylogenetic tree of stony and soft coral AOS-LOX and HPL-LOX fusion proteins. The maximum-likelihood tree was created by Lasergen MegAlign (DNASTAR, Inc., Madison, WI, USA) Full-length sequences of *A. digitifera* (NCBI ID: XM_015915687.1, aug_v2a.10359.t1; XM_015912609.1, and XM_015912608.1), *Orbicella faveolata* (XP_020618718.1, XP_020618720.1, XP_020628673.1, XP_020628674.1, XP_020618715.1, and XP_020618807.1), sea anemone *Exaiptasia pallida* (XP_020897227.1), *G. fruticosa* (EU082210.1 and personal data), *C. imbricata* (KF000373 and KF000374), *P. homomalla* (AF003692.1), and *C. viridis* (AB188528.1) were aligned with *A. millepora* (database ID: c001949, c002203, c002895, c002903). Acronyms as in Figure 1. (**A**) stony corals clade I; (**B**) stony corals clade II; and (**C**) soft coral AOS-LOXs.

To determine the presence of different LOXs in *Acropora* sp., the National Center for Biotechnological Information (NCBI) sequence database survey was conducted. In total, 59 predicted LOX mRNA sequences of *A. digitifera* were found. According to the database annotation, 12 of them were predicted as AOS-LOX (sequence lengths varied between 1201 and 3600 bp), six as 5-LOX (781–2281 bp), one 15-LOX (1261 bp), and one 9*S*-linoleate (1209 bp). The rest of the sequences were predicted to encode other proteins, which was manually confirmed by multiple sequence alignment (MSA). Sequences containing the conserved WLLAK motive and the C-terminal end of LOX (XM_015907419.1, XM_015907422.1, XM_015908627.1, XM_015911043.1, XM_015912608.1, XM_015912609.1, XM_015915687.1, XM_015919244.1, and XM_015924159.1) were included in the MSA of LOXs and the phylogenetic tree was created (Figure 1). The genome database of *A. digitifera* revealed 24 predicted LOXs, but, based on the presence of conserved motives of LOX, only 10 of the sequences (sequence lengths 909–3143 bp) aug_v2a.21361.t1, aug_v2a.16371.t1, aug_v2a.14976.t1, aug_v2a.14977.t1, aug_v2a.19274.t1, aug_v2a.23404.t1, aug_v2a.14591.t1, aug_v2a.08343.t1, aug_v2a.00464.t1, and aug_v2a.10359.t1 were found to be similar to those of LOXs. According to the amino acid (aa) sequence analysis, aug_v2a.08343.t1, aug_v2a.00464.t1, and aug_v2a.10359.t1 were found to be identical with previously retrieved LOX and AOS-LOX sequences, respectively. Short, partial LOX sequences were excluded and only the full-length AOS-LOX sequence (aug_v2a.10359.t1) was used in further studies (Figure 2).

The transcriptome Shotgun Assembly (TSA) library of *A. millepora* at NCBI was also examined and 48 partial predicted LOXs (152–1751 bp) and 18 partial predicted AOSs (203–1439 bp) were detected. Forty-four out of the 48 sequences and eight of the 18 corresponded to the known LOX and AOS sequences, respectively. The retrieved partial LOX sequences of *A. millepora* from the TSA library were too short to be included in MSAs.

Publicly available stony coral transcriptome datasets were searched in parallel. Predicted AOS-LOX and LOX sequences were retrieved from *A. millepora* (27 sequences, 553–4644 bp, four partial LOX, and five full-length AOS-LOXs were used for further analysis), *A. hyacinthus* (19 sequences, 135–1040 bp, all were too short to be included in the LOX analysis), *A. tenuis* (26 sequences, 107–2221 bp, two partial LOXs were used), *Porites astreoides* (25 sequences, 195–1378 bp, one partial LOX was used) and *Anthopleura elegantissima* (out of the 117 retrieved sequences 36 were annotated as LOXs, 219–4116 bp, and four partial LOXs were used). The predicted partial LOX and full-length AOS-LOX sequences of stony corals were aligned with those of known soft coral and mammalian LOXs (Figure 1) and soft coral AOS-LOXs, respectively (Figure 2).

Partial sequences for multiple sequence analysis were selected to include conserved LOX motives: WLLAK, YRDD, HAAVN, and the C-terminus of the LOX sequence (PNGTAI in soft coral AOS-LOXs). Those partial sequences contained all iron-coordinating residues, His 757, His 762, His 943, and Asn 947 (numbering according to *Gersemia fruticosa* AOS-LOX, EU082210.1) and the C-terminal residue of LOXs, necessary for the catalytic activity (Figure S1). In total, 28 stony coral, eight soft coral, and 16 mammalian partial LOXs were aligned and analyzed (Figure 1). The first conserved His was replaced by Lys and Tyr only in *A. millepora* c006594 and *A. tenuis* isotig09255 LOX sequences, respectively (Figure S1). The second and third His were conserved in all the LOX sequences analyzed. Also the fourth iron-coordinating residue, Asn, was conserved in stony and soft coral LOXs with the only exception of *A. millepora* c006594 (Asn substituted by Thr) (Figure S1). Mammalian LOXs have either Asn or His or Ser in this position (Figure S1). Most of the stony coral LOXs ended with Ile or Thr, with the only exception of *A. elegantissima* LOX (comp4343, Ser) (Figure S1). In mammalian and soft coral sequences the final amino acid was conserved as Ile, while as an exception, only *P. homomalla* 8*R*-LOX contained Thr (Figure S1). This soft coral sequence also aligned with stony coral LOXs (Figure 1). The Coffa determinant, an amino acid responsible for *R*/*S* stereospecificity of LOXs [48], was mainly found to be Gly, predicting LOXs with *R*-stereospecificity (Figure S1). However, in *A. millepora* c003910, *A. tenuis* isotig04781, and *A. elegantissima* comp24261_c1_seq5 sequences the amino acid determinant was Ala, predicting LOXs with *S*-stereospecificity. In addition, Ile in *A. millepora* c017280, c001949, c002203, and *A. tenuis* isotig09255; Phe in *A. millepora* c006594, and Val in *A. digitifera* XM_015911043.1 were found (Figure S1).

Mammalian LOXs represented a distinct branch in the MSA analysis (Figure 1, red), with a sequence identity of 40–72% between them. The identity was higher within specific LOX groups (e.g., 5-LOX 93–97%). Soft coral AOS-LOXs (identity 85–100%) were aligned with two groups of stony coral sequences. The first group contained *E. pallida*, *A. tenuis*, and *A. millepora* LOX sequences and two *A. digitifera*, and two *A. millepora* full-length AOS-LOX sequences (identity 52–100%). The identity between stony and soft coral AOS-LOX/LOXs was 47–55% (see a detailed description of the full-length AOS-LOX sequence analysis in Section 2.1.2). The second group containing *G. fruticosa* 11*R*-LOX included four LOX sequences of *A. digitifera*, two AOS-LOX sequences of *A. millepora*, and one LOX sequence of *P. astreoides* (identity 52–99%) (Figure 1). In addition, sequences of *A. millepora*, *A. tenuis* and *A. elegantissima* (identity 78–99%) were paired with mammalian and soft coral (AOS)-LOXs (identity 31–35% and 34–38%, respectively). The remaining ten retrieved stony coral LOX sequences formed a separate clade (identity 46–99%), with *A. millepora* c006594 as an outlier (Figure 1).

Based on the sequence analysis of genomic-transcriptomic data, many LOX isoforms are predicted to be present in stony corals, e.g., 5-LOX, AOS-LOX, etc. Still, according to our current knowledge, there is no defined sequence motive to neither describe nor predict the specificity of LOXs. Moreover, stony coral LOXs are divergent and not a single stony coral LOX has been characterized. Thus, the predictive value of LOX specificity determined by a formal sequence analysis is close to zero. In our sequence analysis, the predicted LOX isoforms did not form a separate group either. In summary, LOXs of interest need to be expressed and analyzed for the product formation in order to confirm the catalytic activity and define specificity.

#### 2.1.2. Fusion Proteins

The NCBI database basic local alignment search tool (BLASTp) search using separate LOX and AOS domains of *C. imbricata* AOS-LOXa retrieved several soft coral AOS-LOX sequences (*C. imbricata*, *P. homomalla*, *G. fruticosa*, and *Clavularia viridis)*, as well as predicted AOS-LOXs from stony corals. In total, only three predicted AOS-LOX mRNA sequences from *A. digitifera* (XM_015915687.1; XM_015912609.1 and XM_015912608.1), six sequences from *Orbicella faveolata* (previously *Montastraea faveolata*, XP_020618718.1, XP_020618720.1, XP_020628673.1, XP_020628674.1, XP_020618715.1, and XP_020618807.1), and one sequence from sea anemone *Exaiptasia pallida* (XP_020897227.1) were almost as long as the full sequences of soft coral AOS-LOXs. Four *A. millepora* AOS-LOXs (database ID: c001949, c002203, c002895, c002903) obtained from a public database were also included in the analysis. The length of soft coral AOS-LOX sequences were 1066–1067 aa and that of stony coral sequences mostly between 1037 and 1081 aa. The shortest sequence, 810 aa and the longest one 1156 aa, corresponded to *O. faveolata* XP_020618807.1 and XP_020628673, respectively. The obtained fifteen stony and six soft coral AOS-LOXs represented distinct branches in the MSA analysis (Figure 2). Stony coral AOS-LOX sequences were further divided into two groups. Over-all, the sequence identity of stony coral AOS-LOXs varied between 41% and 96%, within subgroups from 48% to 73% (excluding highly similar sequences) and from 64% to 88% (Figure 2A,B, respectively), while the sequence identity of soft coral AOS-LOXs, including HPL-LOX, was between 83% and 89% (excluding highly similar sequences of *G. fruticosa*, 99%) (Figure 2C).

Stony corals belonging to different orders (*Actiniaria*/*Scleractinia*, NCBI taxonomy) did not form distinct clades on the sequence analysis of their partial LOXs (Figure 1). A similar result was observed with full-length AOS-LOXs. For example, sea anemone *Exaiptasia pallida* belongs to a different order (*Actiniaria*); still, its AOS-LOX aligns with *O. faveolata*, *A. digitifera*, and *A. millepora (Scleractinia)* AOS-LOXs (Figure 2A). There is no sequence data available on *G. fasicularis* dioxygenases, but *G. fasicularis* belongs to the same suborder (*Faviina*) with *O. faveolata* which AOS-LOX sequences were intermingled with those of other stony coral AOS-LOXs and did not form a separate branch (Figure 2A,B).

All of the predicted AOS-LOX sequences contained the catalytically important amino acids of LOXs: H757, H762, H943, N947, and I1066 (numbering according to *G. fruticosa* AOS-LOX (EU082210.1). Only the C-terminal end of the LOX sequence, including the final iron-coordinating Ile of LOX, was missing from the shortest sequence XP_020618807.1 of *O. faveolata*. Most catalytically important amino acids of AOS, i.e., T66, H67, R349, and Y353, were conserved in stony coral AOS-LOXs. Only T66 to D and R349 to Q in *A. millepora* c002903, and T66 to G and R349 to Q in *A. digitifera* XM_015912608.1 sequences were altered. In addition, T66 and H67 were missing and a change from Y353 to L was observed in the *O. faveolata* XP_020618715.1 sequence. In the multiple sequence analysis these sequences aligned together and formed a separate clade (Figure 2B). In most of the sequences, the Coffa determinant was found to be Gly. In *A. millepora* c001949 and c002203, and *O. faveolata* XP_020618718.1 and XP_020618720.1 the amino acid was replaced by Ile and Leu, respectively. In addition, *O. faveolata* XP_020618715 contained Val in this position. How these alterations affect the chirality of synthesis products is unknown.

In summary, as a result of our analysis we could predict that at least two different AOS-LOXs isoforms would be present in *A. millepora* and *O. faveolata*, and one in *E. pallida*. Whether the predicted isoforms are functionally active and convert polyunsaturated fatty acids or other substrates into the corresponding products remains unknown.

#### 2.1.3. Cyclooxygenases

The BLASTp search using the *P. homomalla* 15*S*-specific COX sequence retrieved COXs from soft corals and predicted non-vertebrate COXs (e.g., from *Crassostrea gigas*, bivalves) (Figure S2). The genomic data about *A. digitifera* for COXs were analyzed in parallel and no sequences with significant similarity to COXs were found. The sequence analysis confirmed that LOX and/or AOS-LOXs were present in both soft and stony corals. At the same time, based on the sequence data available on *A. millepora* and *A. digitifera*, stony corals lacked the COX gene. Currently, there is no sequence data available about *G. fascicularis*.

### 2.2. Eicosanoid Profiling

**Figure 3:**
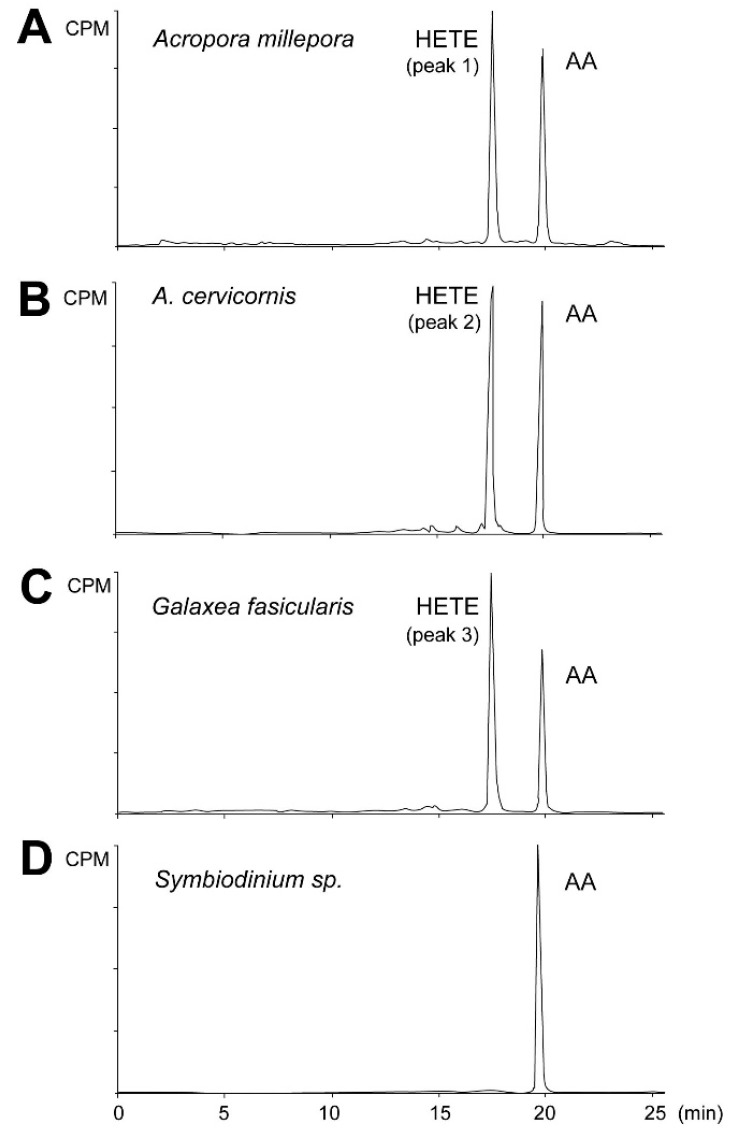
RP-HPLC analysis of incubation products of coral and algal tissue homogenates. Radiochromatograms of the products formed from [1-^14^C] AA by *A. millepora* (**A**), *A. cervicornis* (**B**), *G. fascicularis* (**C**), and *Symbiodinium* sp. isolated from *G. fascicularis* (**D**). Similar results were obtained with *Symbiodinium* sp. samples isolated from both *Acropora* species. One chromatogram is representative of three separate samples. AA—arachidonic acid, HETE—hydroxyeicosatetraenoic acid, CPM—counts per minute.

Initially the metabolites formed from AA by stony coral tissue homogenates were determined by incubation with [1-^14^C] AA (Figure 3). All radiolabeled products, i.e., aldehydes, PGs, α-ketol, cyclopentenone, and HETEs were detected and analyzed within a single run. Exogenous AA eluting at 19.9 min was converted by the *A. millepora* tissue homogenate into a single peak eluting at 17.2 min, making 53% of total conversion rate (Figure 3A, peak 1). A similar result was obtained with the *A. digitifera* sample (Figure 3B, peak 2). Similarly, the tissue homogenate of *G. fascicularis* converted exogenous AA into one peak eluting at 17.2 min, accounting for 60% of total conversion rate (Figure 3C, peak 3). Next, the cells of symbiotic algae were extracted from the tested coral species to detect the dioxygenase activity of *Symbiodinium* sp. Exogenous AA was not converted by any of the algal samples used (Figure 3D).

**Figure 4:**
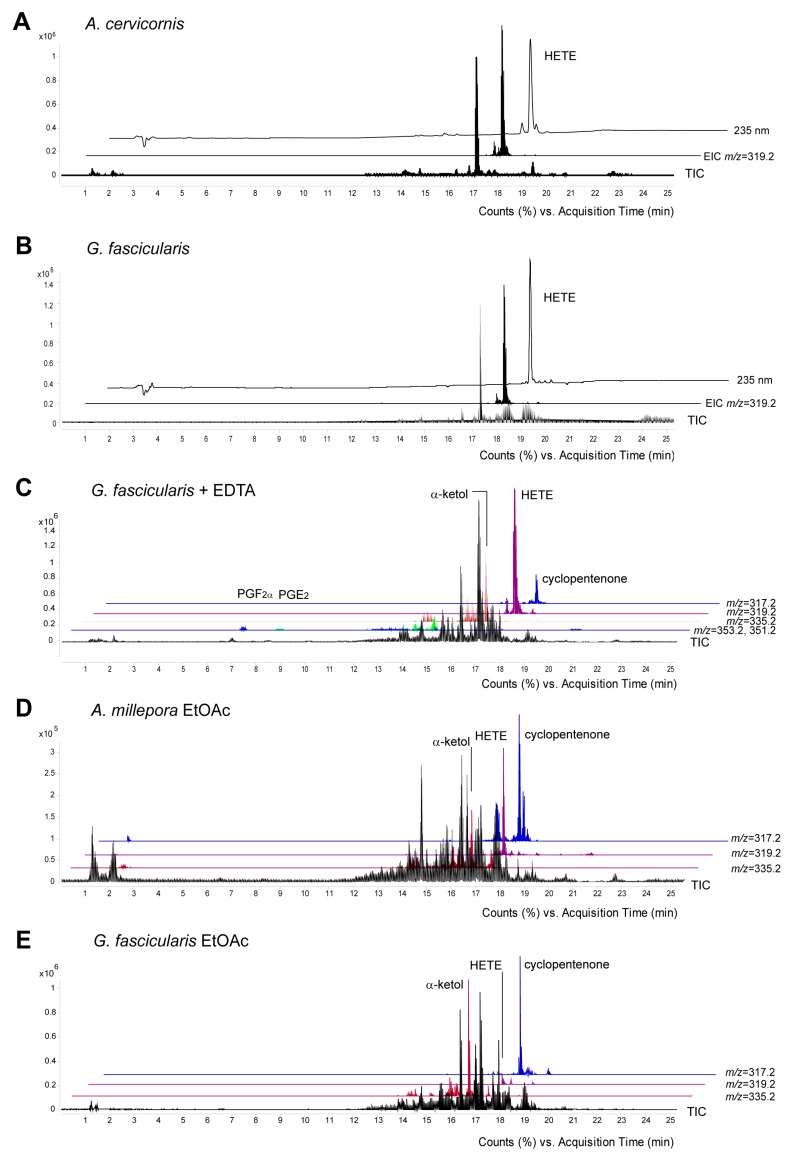
RP-HPLC/MSMS analysis of endogenous metabolites formed by stony corals. Identification of the incubation products formed from [1-^14^C] AA by *A. cervicornis* (**A**), *G. fascicularis* (**B**), and *G. fascicularis* tissue homogenate in the presence of EDTA (**C**). Endogenous eicosanoids were detected in the EtOAc extracts of *A. millepora* (**D**) and *G. fascicularis* (**E**). TIC—total ion current, EIC—extracted ion current corresponding to HETE ([M^−^] *m*/*z* = 319.2), α-ketol ([M^−^] *m*/*z* = 335.2), cyclopentenone ([M^−^] *m*/*z* = 317.2) and PGs (PGF_2α_ [M^−^] *m*/*z* = 353.2, PGE_2_ and PGD_2_ [M^−^] *m*/*z* = 351.2). One chromatogram is representative of analysis of three separate samples.

The peak eluting at 17.2 min (Figure 4A–C) was identified as 8-HETE based on its retention time and mass spectrum which were identical with those of *C. imbricata* 8*R*-LOX product as a standard [23]. In all cases 8-LOXs were soluble enzymes as their activity remained in the soluble fraction of 16,000× *g* supernatant. Our work confirmed that active 8-LOXs were present in stony corals. Interestingly, UV at 235 nm indicated a few small peaks before and after the major peak of 8-HETE (Figure 4A, upper trace). According to the MS analysis, the single ion monitoring of 15-, 11-, 8-, and 5-HETE specific daughter ions, the samples also contained trace amounts of 15-, 11-, and 5-HETEs with the retention times of 16.8, 17, and 17.4 min, respectively (Figure S3).

Somewhat unexpectedly, the MS analysis revealed the presence of PGs in the incubation of *G. fascicularis*. Incubations performed in the presence of a chelating agent, ethylenediaminetetraacetic acid (EDTA), for inhibiting the competitive LOX dependent pathways increased the amount of PGs, PGF_2α_ ([M^−^] *m*/*z* = 353.2) and PGE_2_ ([M^−^] *m*/*z* = 351.2) eluting at 6.9 and 8.5 min, respectively (Figure 4C). The identity of compounds was confirmed by their retention times and spectra with were identical with those of authentic standards (Figure S4).

The presence of endogenous eicosanoids was established in the EtOAc extracts of fresh stony coral tissue homogenates. 8-HETE ([M^−^] *m*/*z* = 319.2), as well as the stable end products of AOS-LOXs, α-ketol ([M^−^] *m*/*z* = 335.2) and cyclopentenone ([M^−^] *m*/*z* = 317.2) were detected in the EtOAc extracts of all the coral samples investigated. In contrast to the incubations with exogenous AA, cyclopentenone and 8-HETE were the main metabolites detected in the fresh EtOAc extracts of *A. millepora*. (Figure 4D, upper traces), while cyclopentenone and α-ketol were the main metabolites in *G. fascicularis* (Figure 4E). The endogenous PGs, PGF_2α_ ([M^−^] *m*/*z* = 353.2) and PGE_2_ ([M^−^] *m*/*z* = 351.2) eluting at 6.9 and 8.5 min, respectively, were also detected as minor metabolites in the EtOAc extracts of *G. fascicularis*. In addition, the formation of aldehydes by cHPL(-LOX) in stony corals was also studied. Aldehydes were found in trace amounts only in the fresh EtOAc extracts of *A. millepora*.

## 3. Discussion

Many eicosanoid related pathways have been proposed to be present in stony corals [38,39]. In this work, the sequence analysis of dioxygenases and the lipid mediator profiling of three stony corals, *A. millepora*, *A. cervicornis*, and *G. fascicularis*, were performed. Based on the sequence data obtained, *Acropora* sp. contained LOXs, AOS-LOXs, and lacked COXs. The detected eicosanoid profiles of *Acropora* sp., presence of HETEs, α-ketol and cyclopentenone, as well as the lack of PGs, were found to be in accordance with the sequence data. At the same time, the detection of PGs next to the major product 8-HETE in a closely-related species, *G. fascicularis*, points at the presence of COX and the metabolic diversity among stony coral species. A scheme of the AA cascade in corals is depicted on (Figure S5).

The discovered diversity between eicosanoid biosynthesis routes in stony corals is not surprising as the biosynthetic ability of soft coral COXs/LOXs to convert AA in vivo and in vitro also varies. For instance, while *P. homomalla* contains a considerable amount of PGs, during biosynthesis with exogenous AA no PGs are formed [13,49]. At the same time, besides the PGs detected as free acids in the coral extracts of *G. fruticosa*, the coral homogenate biosynthesizes PGs in vitro [14,17]. Still, conclusive evidence shows that COX enzymes are responsible for PG synthesis in both species [18,19]. In comparison, in the soft coral *C. imbricata* neither endogenous PGs nor COX activity have been recorded [26].

The proposed synthesis of leukotrienes present in *Pocillopora damicornis* [38] requires 5-LOX activity [50]. In mammals, pro-inflammatory leukotrienes are produced by leukocytes and other immune-reactive cells [50]. Corals contain immune cells [51] which, based on functional similarity, might contain 5-LOX. Indeed, we recorded trace amounts of 15-, 11-, and 5-HETEs in both *Acropora* species. In principle, the presence of 5-HETE indicated that the biosynthesis of LTs in stony corals could be possible, still, the formation of LTs was not confirmed in our study.

The oxygenation specificity of a LOX cannot be predicted based on its primary structure only. Thus, further studies, e.g., the expression and characterization of specific LOX isoforms, are needed to evaluate the properties and specificity of different lipoxygenases present in stony corals. The formation of eicosanoids increases in response to abiotic stress in soft corals [25,26], and eicosanoid producing pathways are up-regulated in response to environmental stress and disease in stony corals [38,39]. Although the predominant biosynthesis product of stony coral tissue homogenates identified in this study was as 8-HETE, it would be intriguing to specify the spectrum of metabolites synthesized under stressful conditions and to determine how they are involved in coral stress response and survival.

In conclusion, the results obtained in this study confirm that the eicosanoid biosynthesis in stony corals is species-specific.

## 4. Materials and Methods

### 4.1. Materials

AA and the PG standard were purchased from Cayman Chemical Co., [1-^14^C] AA from GE Healthcare, and phenylmethylsulfonyl fluoride (PMSF), stannous chloride, and Na_2_SO_4_ from Sigma-Aldrich. Tris, NaCl, and CaCl_2_ were purchased from Merck. Only HPLC grade solvents (Sigma-Aldrich, Taufkirchen, Germany) were used.

### 4.2. Corals

Colonies of *A. millepora*, *A. cervicornis*, and *G. fascicularis* were purchased from a commercial source (aquarium store Kalake, Tallinn, Estonia) and cultivated in a closed-circuit marine aquarium in the Department of Chemistry and Biotechnology at Tallinn University of Technology at an ambient seawater temperature of 25.5 ± 0.2 °C, 35 ppt salinity, under a periodic day-night cycle (12–12 h) and 20% biweekly water exchange.

### 4.3. Sequence Analysis

The sequence search of stony coral dioxygenases was performed using the NCBI database BLASTp [52], the TSA library of *A. millepora* and the searchable genome database of *A. digitifera* [53]. Specifically, to maximize the probability of discovering the divergent AOS-LOX, LOX, and COX homologs from stony corals, BLASTp searches were performed utilizing separate AOS (aa 1–373) and LOX (374–1066) domains of AOS-LOX fusion proteins of *Capnella imbricata* and *P. homomalla* (GenBank accession numbers KF000373.1 and AF003692.1, respectively), and *P. homomalla* COX (AAF93169.1) sequences as queries. In addition, a search using relevant keywords, i.e., lipoxygenase, LOX, allene-oxide synthase, AOS-LOX, cyclooxygenase, COX, PGHS, and arachidonic acid, was conducted in parallel. Publicly available datasets of *A. millepora* [30], *Porites astreoides* [54], *A. hyacinthus*, *A. tenuis*, available at the link of reference [55], and *Anthopleura elegantissima*, available at at the link of reference [56], were searched using keywords lipoxygenase and cyclooxygenase. The obtained DNA sequences were translated by a tool available at the link of reference [57], analyzed and aligned by DNAStar7.1 programs (Lasergen, v7.1, DNASTAR, Inc., Madison, WI, USA). In detail, the retrieved sequences were aligned with the sequences of known soft coral AOS-LOX, soft coral and mammalian LOX or COX by Megalign, Clustal W algorithm (Lasergen, v7.1, DNASTAR, Inc., Madison, WI, USA). The maximum-likelihood phylogenetic trees were created by the same program. Partial LOX sequences were trimmed from conserved WLLAK to the C-terminus of a sequence using EditSeq (Lasergen, v7.1, DNASTAR, Inc., Madison, WI, USA), and realigned. Only full-length sequences were employed for the MSA of coral AOS-LOX and COX.

### 4.4. Preparation of Samples

The enzymatic activity of the tissue homogenates of corals was estimated by in vitro incubations as described previously [26]. In a standard assay, the coral tissue (0.33 g mL^−1^) was homogenized (Tissue Tearor, set 5) in 50 mM Tris-HCl pH 8.0 buffer containing 0.5 mM PMSF on ice. Immediately, an aliquot of the homogenate (6.6 mg) was incubated with 50 μM [1-^14^C] AA (GE Healthcare) in 1 mL (final volume) of 50 mM Tris-HCl pH 8.0, 100 mM NaCl, and 1 mM CaCl_2_ pH 8.0 at room temperature, with constant stirring for 5 min. Incubations in 50 mM Tris-HCl, 50 mM EDTA at pH 8.0 were conducted in order to inhibit the prominent LOX activity. The reactions were terminated with SnCl_2_ (10 mM) and, after acidification with HCl to pH 3.5, the products were extracted with ethyl acetate. The extract was dried over Na_2_SO_4_, evaporated to dryness and re-dissolved in a 4:1 methanol: water solution for further product analysis by reverse-phase high-performance liquid chromatography (RP-HPLC) In parallel, to analyze endogenous eicosanoids, the fresh coral homogenates were extracted with ethyl acetate (EtOAc) and dried over Na_2_SO_4_ [25]. The concentrated extracts (0.1 mg mL^−1^) were stored at −20 °C. These procedures were conducted with three independent coral branches and each of them was used separately for eicosanoid analysis.

### 4.5. Isolation of Zooxanthellae Cells

Coral tissue was homogenized (Tissue Tearor, set 5). Zooxanthellae *(Symbiodinium* sp.*)* were extracted from the tissue homogenate by centrifugation at 4000× *g*, algal cell pellets were washed in 5–10 mL of fresh filtered seawater and re-pelleted three times [58]. Sample homogeneity was controlled by a microscope, at 400× magnification (Nikon, Tallinn, Estonia). The cell pellets were weighted and stored at −80 °C. In a standard assay, the algae cell pellet (170 mg mL^−1^) in 50 mM Tris-HCl pH 8.0 buffer containing 0.5 mM PMSF was sonicated 3 × 5 s (Ultrasonic Cell Disruptior/Cole Parmer, Vernon Hill, IL, USA) on ice. Incubations with 50 μM [1-^14^C] AA were conducted as described above and analyzed.

### 4.6. RP-HPLC

The samples were analyzed using a Zorbax Eclipse XDB-C18 column (5 µm, 4.6 × 150 mm), run on the Agilent 1200 Series HPLC system (Santa Clara, CA, USA), connected to a diode array detector, followed by the 500TR Series Flow Scintillation Analyzer (Packard Bioscience, Meriden, CT, USA) or the Agilent 6540 UHD Accurate Quadrupole Time-of-Flight MS/MS with an Agilent Jet Stream™ ESI source in negative mode(Santa Clara, CA, USA). The elution was performed with a solvent system of ACN/water/formic acid (98.9%/1.0%/0.1% *v*/*v*/*v*) (A) and water/formic acid (99.9%/0.1% *v*/*v*) (B), 0–8 min isocratic (35%A:65%B), 9–17 min gradient to 100% A, 18–30 min 100% A at a flow rate of 1 mL min^−1^ [26]. The data were analyzed by Agilent MassHunter Workstation Software Qualitative Analysis, Version B.05.00 Build 5.0.519.0. Eicosanoids were identified by comparing their retention times and mass spectra with those of authentic standards.

## Supplementary Materials

The following are available online at www.mdpi.com/1660-3397/16/1/10/s1; Figure S1: Multiple sequence alignment of partial coral and mammalian LOXs, Figure S2: Phylogenetic tree of COX sequences created by BLASTp search using *P. homomalla* 15*S*-COX sequence as a query, Figure S3: Identification of 15-, 11-, 8-, and 5-HETEs formed by *A. cervicornis*, Figure S4: Identification of prostaglandins formed by *G. fascicularis*, Figure S5: AA cascade in corals.
