# Supplementary material for: Eicosanoid Diversity of Stony Corals
Source: Mar Drugs. 2018 Jan 3;16(1):10. doi: 10.3390/md16010010 (PMC5793058; doi:10.3390/md16010010)
Supplement: Supplementary file 1 [file marinedrugs-16-00010-s001.docx]

# **Supplementary materials**


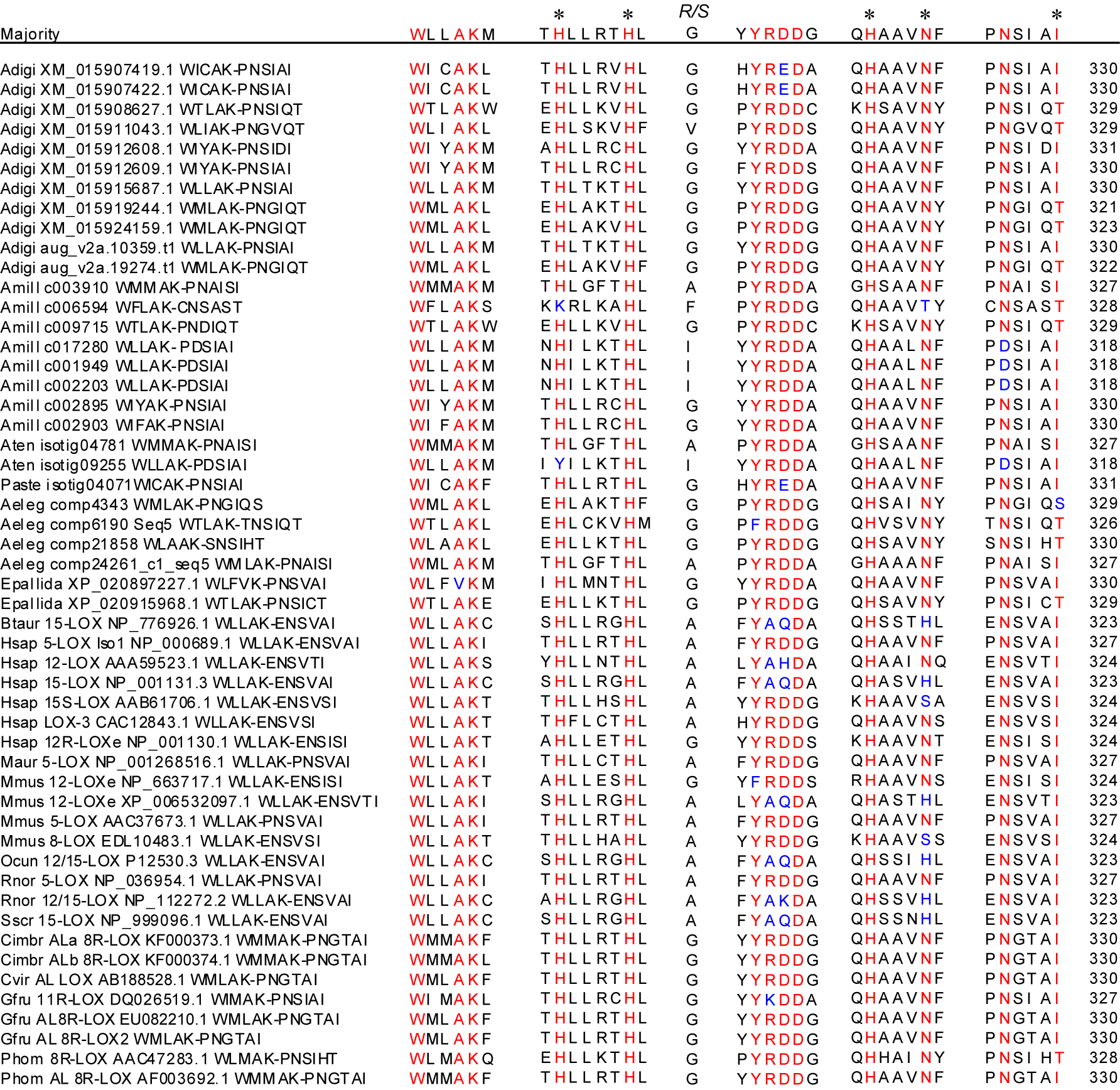


**Figure S1. Multiple sequence alignment of partial coral and mammalian LOXs.** Only sequences containing WLLAK to PNSIAI (about 318-330 aa) were included in the analysis. **Stony coral LOXs:** *A. digitifera,* Adigi: XM_015907419.1, XM_015907422.1, XM_015908627.1, XM_015911043.1, XM_015912608.1, XM_015912609.1, XM_015915687.1, XM_015919244.1, XM_015924159.1, aug_v2a.10359.t1 and aug_v2a.19274.t1; *A. millepora,* Amill: c017280, c003910, c009715, c006594, full-length AOS-LOXs: c002895, c002903, c001949 and c002203; *A. tenuis,* Aten: isotig04781 and isotig09255; *P. asteroides*, Paste: isotig04071; *Anthopleura elegantissima,* Aeleg: comp4343, comp6190_c0_seq5, comp21858, comp24261_c1_seq5; *Exaiptasia pallida,* Epallida: XP_020897227.1 and XP_020915968.1. **Soft coral LOXs:** *G. fruticosa*, Gfru: AOS-8*R*-LOX and AOS-8*R*-LOX2 (EU082210.1 and personal data), 11*R*-LOX (DQ026519.1); *C. imbricata,* Cimb: AOS-8*R*-LOXa and HPL-8*R*-LOX (KF000373 and KF000374); *P. homomalla,* Phom: AOS-8*R*-LOX (AF003692.1) and 8*R*-LOX (AAC47283.1), and *Clavularia viridis,* Cvir: putative AOS-LOX (AB188528.1). **Mammalian LOXs:** *Bos taurus*, Btaur: 15-LOX (NP_776926.1); *Homo sapiens*, Hsap: 5-LOX (NP_000689.1), 12-LOX(AAA59523.1), 15-LOX (NP_001131.3), 15S-LOX(AAB61706.1), LOX-3 (CAC12843), 12*R*-LOXe (NP_001130.1); *Mesocricetus auratus,* Maur: 5-LOX (NP_001268516.1); *Mus musculus*, Mmus: 12*R*-LOXe (NP_663717.1), 12/15-LOX XP_006532097.1), 5-LOX (AAC37673.1), 8-LOX (EDL10483.1), *Oryctolagus cuniculus*, Ocun: 12/15-LOX (P12530.3), *Rattus norvegicus,* Rnor: 5-LOX (NP_036954.1), 12/15-LOX (NP_112272.2), *Sus scrofa*, Sscr: 15-LOX (NP_999096.1). Red – conserved residues, blue – alterations, asterisk – conserved iron-coordinating aa, *R/S* – Coffa determinant.

**Figure S2. Phylogenetic tree of COX sequences created by BLASTp search using P. homomalla 15S-COX sequence as a query. The COX sequence used for search is highlighted in yellow.**

**Figure S3. Identification of 15-, 11-, 8-, and 5-HETEs formed by *A. cervicornis*.** Traces: 235 nm (purple); 15-HETE specific daughter ion *m/z*=175 (green); 11-HETE *m/z*=165 (red); 8-HETE *m/z*=165 (black); 5-HETE *m/z*=115 (blue).

**
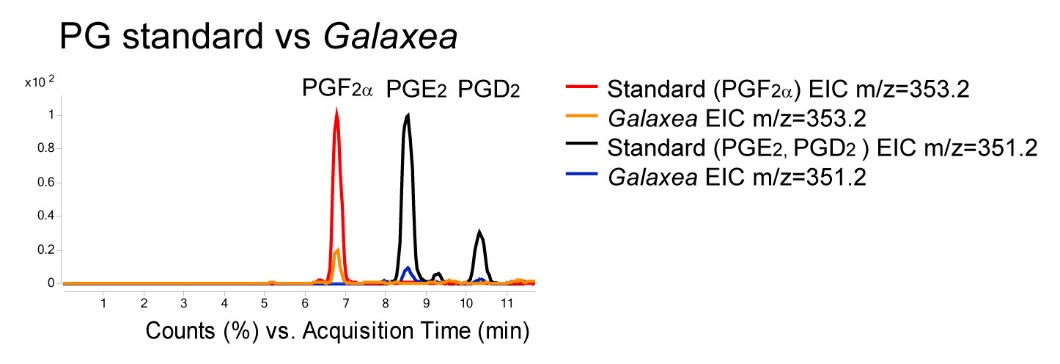
**

**Figure S4. Identification of prostaglandins formed by *G.* *fascicularis*.** EIC – extracted ion current corresponding to PGF_2α_ ([M^-^] *m/z* = 353.2), PGE_2_ and PGD_2_ ([M^-^] *m/z* = 351.2).

 **Figure S5. AA cascade in corals. AOS-LOX pathway is depicted in red. Enzymes are encircled. HPETE- hydroperoxyeicosatetraenoic acid.**
